# Support Vector Machines: A Literature Review on Their Application in Analyzing Mass Data for Public Health

**DOI:** 10.7759/cureus.77169

**Published:** 2025-01-08

**Authors:** Khyathi G, Indumathi K P, Jumana Hasin A, Lisa Flavin Jency M, Sibyl Siluvai, Krishnaprakash G

**Affiliations:** 1 Public Health Dentistry, Sri Ramaswamy Memorial (SRM) Kattankulathur Dental College and Hospital, SRM Institute of Science and Technology, Chennai, IND

**Keywords:** applications, data, patient management, public health, support vector machine

## Abstract

This study considers the literature on support vector machines (SVMs) in the area of public health data analysis, particularly evaluating their ability to harness big data for disease classification and health predictions. SVMs have been remarkably embraced for two decades in clinical diagnosis, patient management, and prediction of health trends owing to their high precision and robustness. This review suggests the ability of the method to support spatially relevant health system responses through the assessment of SVM advantages and disadvantages in public health and future research agendas, including improving scalability, integrating SVMs with emerging data sources like the Internet of Things (IoT) and genomic data, and enhancing model transparency to support real-world public health decision-making.

## Introduction and background

A support vector machine (SVM) is a supervised machine learning technique developed in the early 1990s. It is commonly used in classification and regression tasks due to its efficiency and robustness in dealing with large data [1]. It helps classify the given data into different classes and targets to augment the margin between them by determining the best possible hyperplane for that model to make accurate predictions [2]. SVMs can also deal with non-linear mapping by transforming the data to higher dimensions by adopting kernel functions [3]. Due to their high degree of flexibility, SVMs can explore complex data structures in many fields of study including but not limited to public health.

Data indicates the enormously complex and many sources of domain information that are produced by, for example, health fitness devices, social networking, electronic patient files, systems for health monitoring, and so on [4]. It is this data that usually possesses high volume, high velocity, and high variety, which poses questions and answers alongside opportunities and challenges for data analytics [5]. In this regard, mass data is fundamental in public health as it provides a great deal of information on the behavior of diseases, health behaviors, and general health of the increasing population. For instance, electronic medical records include data on the risk factors and treatment outcomes of each of the patients, which is all-inclusive, while patient surveillance goes to social networking and helps to monitor the spread of epidemics literally as they happen [6].

When it comes to public health, SVM and mass data converge upon the application of SVM algorithms in the classification, prediction, and pattern analysis of such datasets, enabling faster and better decision-making. SVMs are also excellent for extracting significant information from rich health datasets since they accommodate multiple forms of data, such as text, numbers, and images, contained in large datasets [7].

SVM models can predict the risk of chronic conditions, aid in targeted care planning, and even ascertain possible events, such as disease outbreaks, through patterns observable in big data. There is a positive outcome in the interaction between SVMs and big data, whereby public health systems can respond to health needs proactively, manage resources effectively, and improve health status among populations [8]. The particular emphasis of this paper is on the review of SVMs used and their various applications, including forecasting morbidity and mortality, managing chronic conditions, and addressing personalized healthcare.

## Review

Methodology

A comprehensive search was undertaken using Pubmed, Scopus, and Web of Science to conduct this review. Keywords employed included "support vector machine (SVM)", "machine learning", "public health analytics", "chronic disease prediction", "artificial intelligence in healthcare", "patient management", and "data and applications". Owing to the limited availability of research related to the subject of interest, existing literature between 1995 and 2020 was included in the review. Search results were improved by reading the bibliographies of the articles found. This ensured that the topic of SVMs in analyzing mass data for public health was thoroughly explored.

Inclusion Criteria

(i) SVM applications in public health: Papers discussing the use of SVMs in detecting diseases, predicting epidemics, and managing healthcare data.

(ii) Disease monitoring and predictive analyses: That is, research focusing on applications of SVMs to track infectious diseases, manage chronic conditions, and develop individualized medicine.

(iii) Clinical and non-clinical settings: This includes studies on SVMs in the context of hospitals, emergency care, home health, and public health through informatics, right from mental health monitoring to resource allocation.

(iv) Types of research and publication: Original research, systematic reviews, meta-analytic studies, and case studies from peer-reviewed journals.

(v) Publications published in English only: Only the studies that were available in English were considered.

Exclusion Criteria

(i) Non-healthcare settings: Studies focusing on industrial applications or other non-healthcare-related uses of SVMs, unrelated to public health or clinical practice.

(ii) Insufficient data or non-generalizable results: Research with limited or non-representative data, such as isolated case reports or studies lacking relevance to broader healthcare outcomes.

(iii) Non-peer-reviewed sources: Conference abstracts, opinion articles, or literature not published in peer-reviewed journals.

(iv) Irrelevant applications: Articles addressing SVMs in domains unrelated to healthcare, such as purely computational or theoretical studies without healthcare or public health implications.

Applications of SVMs in public health data analysis

**Figure 1:**
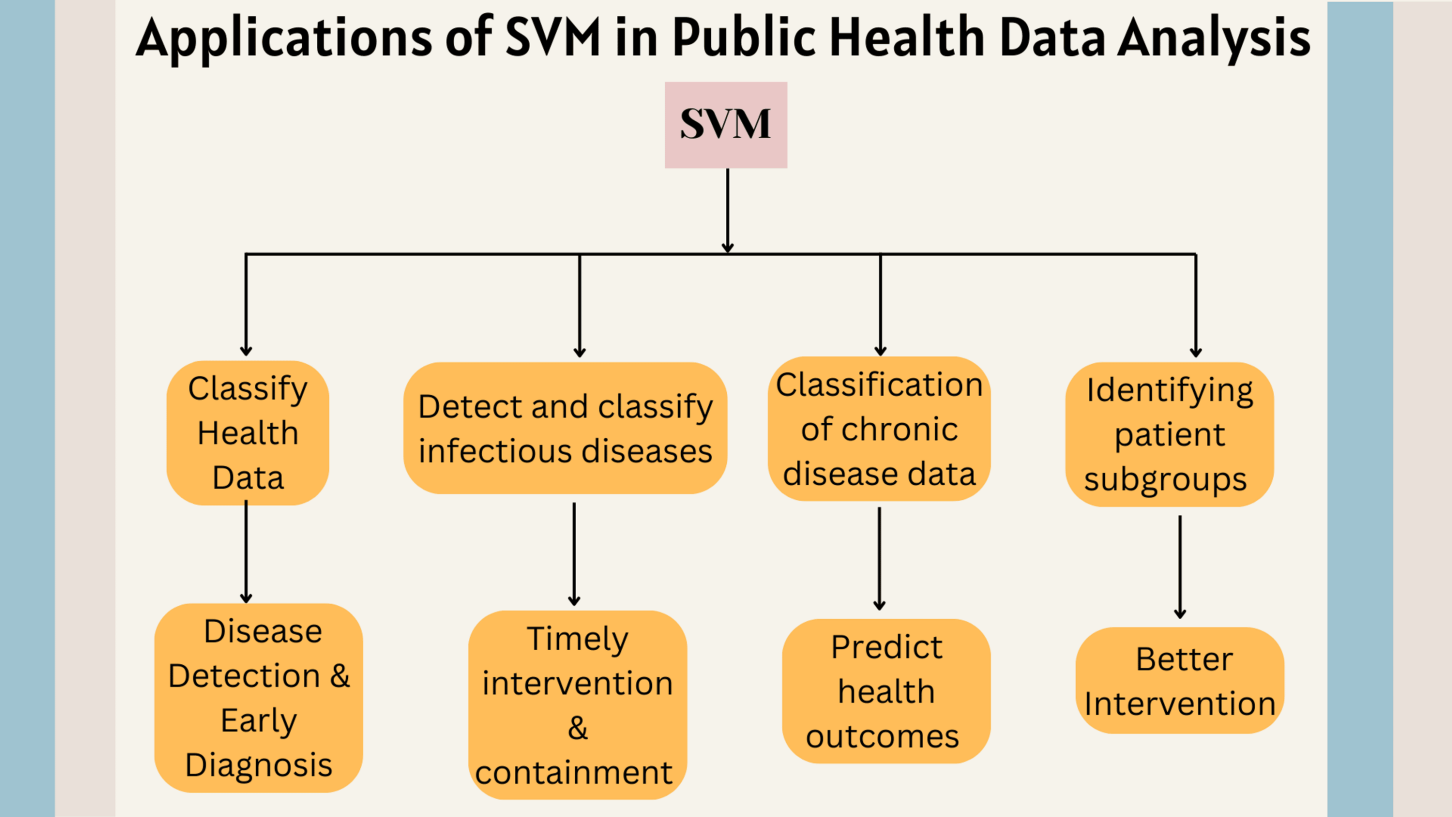
Applications of SVMs in public health data analysis SVM: support vector machine The figure was created by the authors.

Figure 1 illustrates the applications of SVMs in public health data analysis.

The use of SVMs in data analysis of public health primarily involves the detection of disease and epidemics, wherein SVM models are exercised to classify healthcare data for timely assistance [9]. In another instance, SVMs have been applied in studies to detect and even classify disease outbreaks so that control measures can be implemented in time. This is critical especially in epidemic predictions as there exists a huge volume of data that must be scrutinized for possible signs of a viral outbreak for swift intervention by health authorities [10]. In the same vein, SVMs can also be employed for systemically organizing the information of chronic illness patients to predict the future direction of the patient’s health given the records kept. SVMs optimize patient management by enhancing preventive healthcare and resource allocation through identifying patients at high risk and estimating risk factors [11]. Also, SVMs are utilized in personalized medicine. For instance, SVMs allow for the identification of specific characteristics of groups of patients that enhance treatment effectiveness [12].

Disease Detection and Outbreak Prediction

SVMs have been used for forecasting epidemics as well as for the discovery of emerging infectious diseases. They also facilitate monitoring illness trends in advance by processing surveillance, social media, and electronic health record (EHR) data. For instance, during the COVID-19 pandemic, SVMs were crucial for classifying patients into subgroups based on clinical traits such as age, comorbidities, and symptom severity, helping identify at-risk individuals and optimize resource utilization. They also played a key role in predicting trends, supporting diagnosis through imaging, and enabling risk stratification for better resource allocation. SVMs' ability to process high-dimensional data makes them particularly effective in these applications [8]. Similarly, SVMs were used for tracking influenza and predicting the onset of the disease by looking at social media and search data to find behavioral patterns that tie to a new health threat [10]. One key advantage of using SVMs for outbreak prediction is their ability to manage both structured (e.g., EHR data) and unstructured data (e.g., social media posts). By combining these data sources, SVM models can offer a more comprehensive understanding of disease dynamics, enabling quicker responses and more efficient containment strategies [13].

Chronic Disease Prediction and Management

SVMs are frequently applied to predict the onset and progression of chronic diseases such as diabetes, cardiovascular disease, and cancer. These diseases are major public health burdens, and early detection can significantly improve health outcomes. In diabetes management, SVMs have been used to analyze clinical data such as age, body mass index (BMI), blood pressure, and blood glucose levels to predict the risk of diabetes onset, enabling preventive interventions [14]. SVMs have also been utilized in predicting the risk of cardiovascular diseases by analyzing EHRs, including patient demographics, lifestyle factors, and medical history. These predictive models are valuable for identifying high-risk individuals who may benefit from early medical interventions [7].

SVM models have been applied to oncology as well to help predict cancer progression and treatment outcomes. For example, SVMs have been employed to classify breast cancer patients based on histopathological features, genetic data, and clinical profiles, achieving high classification accuracy and improving early diagnosis and treatment planning [2]. Such applications are crucial in personalized medicine, where treatment plans are tailored to the specific characteristics of the patient, improving the effectiveness of therapeutic interventions.

One of the healthcare domains that is using SVMs to its advantage is data science, which, in turn, improves diagnosis, treatment, and preventive measures. In dentistry as well as in those fields employing other imaging diagnostics, SVMs are used to monitor the detection and diagnosis of oral tissue pathologies, such as dental caries, periodontal ailments, and oral cancer, by evaluation of clinical outcomes, images, and biomarkers. SVM models are preventative in nature; through their application, diseases can be detected at earlier stages, thus curbing the progression and, in turn, the costs of healthcare.

In dental radiology, SVMs play an important role in the evaluation of X-rays, cone-beam computed tomography (CBCT) scans, and others to identify dental caries, fractures, and cyst-oriented image data. They assist orthodontists in their prediction of potential risk of tooth movement. In a large population screening to identify risk factors for oral disease, SVMs can help in the epidemiology of dental public health informatics. Additionally, they can aid in predicting the patterns of trends and improving the effectiveness of campaigns solely aimed at the specific populations at risk, such as fluoridation and oral hygiene interventions. SVMs can also help in the analysis of the effects of certain treatments and the success of dental implants. While providing decision-making insights, data quality, and model interpretability, integration within the clinical workflow is also critical. SVMs' advanced techniques enhance the quality of clinical practices and the health of the dental population.

Mental Health Monitoring and Suicide Prevention

Mental health has become a growing focus in public health, and SVMs have shown significant promise in the early detection of mental health disorders. By analyzing various data sources such as social media posts, surveys, and EHRs, SVMs can identify individuals at risk for conditions like depression, anxiety, and suicidal tendencies. For instance, De Choudhury et al. (2013) used SVMs to analyze social media data to predict the onset of depression, identifying behavioral markers that could serve as early warning signs [15]. This approach to mental health monitoring allowed for proactive intervention before the onset of severe symptoms, improving outcomes for individuals at risk. In clinical settings, SVMs have also been used to predict suicide risk by analyzing patient records, including mental health history, demographic data, and behavioral patterns. These predictive models can assist healthcare providers in identifying individuals who may benefit from intensive monitoring or therapeutic interventions [16]. The ability to identify at-risk individuals early and intervene effectively is critical for reducing suicide rates and improving mental health outcomes.

Personalized Medicine and Treatment Recommendations

SVMs play a significant role in personalized medicine, where they are used to predict how individual patients will respond to specific treatments based on their genetic, clinical, and lifestyle data. This is particularly important in areas such as oncology, where patients often respond differently to treatments based on their genetic makeup. By analyzing genomic data, SVMs can help identify cancer patients who are most likely to benefit from certain therapies, allowing for more effective and targeted treatments [11]. SVMs have also been used to analyze pharmacogenomic data to predict how patients will respond to various medications, reducing the likelihood of adverse drug reactions and improving treatment outcomes. For instance, SVM models have been employed to predict the response to chemotherapy based on the molecular characteristics of a tumor, allowing oncologists to choose the most effective drug regimen for a patient [17].

Health Resource Allocation and Cost Prediction

SVMs have also been applied in health resource allocation and cost prediction, assisting public health authorities and healthcare providers in optimizing their resource management. By analyzing patterns in hospital admissions, patient demographics, and medical conditions, SVMs can predict the demand for healthcare services, allowing for more efficient planning and resource allocation. For example, SVMs have been used to predict hospital readmissions, identify patients who are at high risk of returning to the hospital, and enable preventive interventions that reduce readmission rates [18].

Moreover, SVMs have been utilized to forecast healthcare costs, providing valuable insights for budget planning and resource distribution. By analyzing historical data on treatment costs, patient outcomes, and service utilization, SVM models can estimate future healthcare costs, helping organizations allocate resources more effectively and improve cost-efficiency in healthcare delivery [19].

Significance in Public Health Dentistry

In public health dentistry, SVMs play a significant role in advancing data science, which supports healthcare domains like clinical care and public health. SVMs are used to enhance diagnosis, treatment, and prevention by monitoring and diagnosing oral pathologies, such as dental caries, periodontal diseases, and oral cancer, through the evaluation of clinical outcomes, images, and biomarkers. Their preventative nature allows for early disease detection, curbing progression, and reducing healthcare costs.

In dental radiology, SVMs are vital for evaluating X-rays, CBCT scans, and other imaging data to detect dental caries, fractures, and cysts. They also assist orthodontists in predicting the risk of tooth movement. Additionally, SVMs can contribute to large-scale population screenings, identifying risk factors for oral diseases, and supporting dental public health informatics. They can help predict trends, improve targeted campaigns (e.g., fluoridation and oral hygiene interventions), and assess the success of treatments and dental implants. Despite these advantages, ensuring data quality, model interpretability, and integration into clinical workflows remains essential. Overall, SVMs can enhance clinical practice and improve public dental health outcomes.

Advantages of SVMs in public health

SVMs offer several advantages when dealing with public health-related data. Their ability to cope with nonlinear and highly dimensional data, which is the norm in healthcare data due to multiple factors including age, genetic factors, economic status, and clinical evaluation factors, is one of the biggest benefits of SVMs [20]. Due to their superior classification performance, SVMs allow for effective predictive analytics of patient outcomes and the risk of diseases [21]. Also, since health data usually has a lot of turbidity or outliers which might mess up other models, the overfitting tolerance of the algorithm is a positive aspect [22]. Even with complex and heterogeneous datasets, SVMs can maintain performance levels by maximizing the separation between the classes of data. In addition, through kernel functions, SVMs can also project the data into higher dimensional spaces, which allows them to be efficient with all kinds of datasets in the field of public health [23].

Limitations of SVMs in health data analysis

Societal awareness is inherent in the constraints of social processes of communication and interaction relevant to SVMs as a method of conducting social surveys. The model is sensitive to the parameter selection (for instance, the regularization parameters and kernel type), which is a strong disadvantage since it affects the model's performance and applicability [6]. This problem arises because the SVMs have many parameters that must be set in order to achieve the desired results, and for best performance, these parameters must be optimized using complex cross-validation processes. Another limitation of SVMs is the time it takes for a trained model to be built, especially in the case of public health studies and other fields where vast amounts of data are involved in analyzing information [8]. Resources may not be enough since the SVM algorithm requires a lot of storage and processing power, thus making it hard to train an SVM model on such databases in many public health situations. Hence, except in cases where larger implementation will have the luxury of time and moderate computational power, SVMs would be most suited for a few small and well-defined datasets. Integrating SVMs into clinical workflows faces challenges such as the need to train healthcare professionals, improve model explainability, and overcome system integration issues with legacy infrastructure. Data quality, regulatory compliance, and addressing ethical concerns like bias also pose hurdles. Additionally, rigorous clinical validation is essential to build trust. Overcoming these challenges will enhance SVM integration and improve healthcare outcomes.

Future directions

To enhance the predictability and scalability, future works on the use of SVMs in public health would focus on, among other things, augmenting SVMs with other machine learning (ML) techniques, such as neural networks in this case. Hybrid models, which seem to take the best attributes of various approaches, could help solve some weaknesses of SVMs when it comes to issues like sensitivity to parameters and processing overheads. Besides, the field applications of SVMs in monitoring public health data stand potential in that many real-time trends including social media and wearable tech could be used in analyzing them with the aim of forecasting and curbing health problems almost immediately.

The positive impact of SVMs in health research and in informing health policies is, however, likely to go up with advancements in computing power and improved ways of optimizing SVMs. The integration of SVMs along with other ML techniques is also likely to contribute towards solving some of the challenging health problems and improving health within the population as a whole [24].

## Conclusions

SVMs have proved to be particularly useful for examining large and intricate public health data due to their ability to learn and model complex non-linear relationships and handle high-dimensional data. The strength of SVMs also lies in the fact that they allow for accuracy in the analysis of cases of illness prediction and the detection and classification of health status. This technique is a boon in the field of public health where the variation in data quality can be very high since it is inherently robust to noise and class imbalance. However, a few challenges prevent SVMs from being more widely used.

These are the challenges of high computational cost, tuning of parameters, and difficulty in interpreting the outcome. Despite these limitations, increasing possibilities with SVMs are connected to their capability to be mixed up with other techniques, for instance, ensemble methods and deep learning approaches. In line with the arguments mentioned earlier, further research may focus on more practical aspects such as improving the scalability, audiovisual interpretation, and incorporation of the technique for real-time data in public health treatments. Additionally, developments in computational resources and applications are slowly but surely eliminating the limitations associated with SVMs, paving the way for their application in large-scale projects. Overall, SVMs can still be useful in spotting important trends within a large amount of health data and guiding evidence-based practice in public health.
